# Urinary biomarkers for the detection of ovarian cancer: a systematic review

**DOI:** 10.1093/carcin/bgac016

**Published:** 2022-02-15

**Authors:** Gemma L Owens, Chloe E Barr, Holly White, Kelechi Njoku, Emma J Crosbie

**Affiliations:** 1 Division of Cancer Sciences, School of Medical Sciences, Faculty of Biology, Medicine and Health, University of Manchester, St Mary’s Hospital, Manchester M13 9WL, UK; 2 Obstetrics and Gynaecology Department, Manchester University NHS Foundation Trust, Manchester Academic Health Science Centre, Manchester M13 9WL, UK

## Abstract

Currently, the only definitive method for diagnosing ovarian cancer involves histological examination of tissue obtained at time of surgery or by invasive biopsy. Blood has traditionally been the biofluid of choice in ovarian cancer biomarker discovery; however, there has been a growing interest in exploring urinary biomarkers, particularly as it is non-invasive. In this systematic review, we present the diagnostic accuracy of urinary biomarker candidates for the detection of ovarian cancer. A comprehensive literature search was performed using the MEDLINE/PubMed and EMBASE, up to 1 April 2021. All included studies reported the diagnostic accuracy using sensitivity and/or specificity and/or receiver operating characteristics (ROC) curve. Risk of bias and applicability of included studies were assessed using the QUADAS-2 tool. Twenty-seven studies were included in the narrative synthesis. Protein/peptide biomarkers were most commonly described (*n* = 18), with seven studies reporting composite scores of multiple protein-based targets. The most frequently described urinary protein biomarker was HE4 (*n* = 5), with three studies reporting a sensitivity and specificity > 80%. Epigenetic (*n* = 1) and metabolomic/organic compound biomarkers (*n* = 8) were less commonly described. Overall, six studies achieved a sensitivity and specificity of >90% and/or an AUC > 0.9. Evaluation of urinary biomarkers for the detection of ovarian cancer is a dynamic and growing field. Currently, the most promising biomarkers are those that interrogate metabolomic pathways and organic compounds, or quantify multiple proteins. Such biomarkers require external validation in large, prospective observational studies before they can be implemented into clinical practice.

## Graphical Abstract

**Graphical Abstract:**
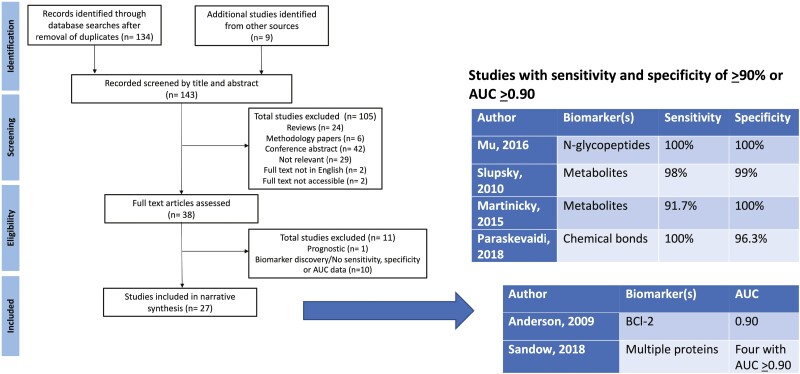

## Introduction

Ovarian cancer is the most lethal gynaecological malignancy, accounting for 4200 deaths in the UK each year (1). Due to a lack of specific symptoms and effective screening strategies, almost 60% of women present with advanced disease (stage III and IV), when the 5-year survival rate is less than 30%. In contrast, women presenting with stage I disease have a 5-year survival rate of >90% (2). The stark disparity in survival between early and late-stage disease has spurred interest in developing novel diagnostic biomarkers that can detect ovarian cancer while it is confined to the ovary.

Currently, histopathological examination is the only definitive method for diagnosing ovarian cancer. Transvaginal ultrasonography (TVS) in combination with serum cancer antigen 125 (CA125) levels is widely utilized in the initial evaluation of suspected cases of ovarian cancer. TVS can identify adnexal masses but is less reliable in differentiating benign from malignant tumours. Furthermore, its diagnostic accuracy is user-dependent and detection of possible metastasises at other sites may be elusive or undetectable until they reach a sufficient size (3). CA125 is the best-characterized serum biomarker for ovarian cancer. CA125 levels are elevated (*>*35 units/ml) in 80% of women with advanced disease, but only 50% of women with early-stage disease (4). Additionally, CA125 lacks specificity as levels can also be elevated in other physiological and pathological conditions, such as menstruation, pregnancy, endometriosis and non-gynaecological cancers (5), making it unreliable for screening and early detection. Other serum biomarkers have been evaluated in the diagnosis of ovarian cancer, either alone or in combination with CA125. Of these, human epididymis 4 (HE4) has been the most promising, although its performance is inferior to CA125 (6). More recently, strategies combining multiple circulating protein biomarkers (7,8) and those utilizing circulating tumour DNA have been developed (9).

Blood has traditionally been used for ovarian cancer biomarker discovery; however, urine harbours a wide variety of molecules with the potential to serve as biomarkers, including excreted proteins, antibodies, RNAs, endogenous metabolites and organic compounds (10). Urine has several key advantages over blood as a source of biomarkers. First, urine is easily accessible, non-invasive, available in unlimited quantities and benefits from strong patient acceptability (11). Second, as a product of homeostasis, urine is likely to be reflective of changes in chemical composition from multiple body sites. Moreover, urine proteins are less complex and more stable than the blood proteome (12). In recent years, numerous urinary biomarkers have been explored in ovarian cancer, including CA125, HE4 and osteopontin. Here, we present a systematic review of the diagnostic accuracy of urinary biomarkers for the detection of ovarian cancer.

## Materials and methods

This systematic review was registered on the International Prospective Register of Systematic Reviews (PROSPERO Registration No.: CRD42020212902) and is reported in accordance with PRISMA (Preferred Reporting Items for Systematic Reviews and Meta-Analyses) guidelines (13).

### Literature search

A comprehensive literature search was performed using the MEDLINE/PubMed and EMBASE databases to identify articles evaluating the diagnostic accuracy of urinary biomarkers for the detection of ovarian cancer. We used the following keywords and MeSH terms: ovar* AND (cancer OR neoplasm) AND (detection OR diagnosis) AND urine AND (biomarker OR biological marker OR assay). The search was performed for articles published from inception until 1 April 2021. The searches were restricted to English language publications. Additional relevant manuscripts were identified by searching reference lists and conference abstracts.

### Study selection

Two authors (CB & KN) independently reviewed abstracts and full-text articles against the pre-specified eligibility criteria. Disagreements regarding inclusion were resolved through discussion with a third reviewer (GO). Studies were included if they met the following criteria: (1) case-control or cohort study of urinary biomarkers; (2) reported the diagnostic capability of urinary biomarkers for the detection of ovarian cancer using both sensitivity and/or specificity and/or area under a ROC curve (AUC). Due to the paucity of publications in this area, we did not set a minimum number of patients/controls in our inclusion criteria. We excluded studies that did not evaluate the diagnostic accuracy of the biomarkers against standard diagnostic methods (histopathology); and studies on the accuracy of prognostic and predictive biomarkers.

### Data extraction and quality assessment

Data were extracted from selected studies by two independent authors (CB & KN), using a standardized form. Any disagreements were resolved by a third reviewer (GO). Extracted data from each full-text manuscript included: study characteristics (author, year of publication, journal, country), study design, assay evaluated, protocol for urine collection, histological type of ovarian cancer, International Federation of Gynecology and Obstetrics (FIGO) staging, definition of the control group, number of participants in the ovarian cancer and control groups, assay cut-off and the diagnostic accuracy of the test (sensitivity, specificity, negative predictive value, positive predictive value, AUC). Where more than one patient cohort were described, the final validation group was used. A 2 × 2 table with numbers of true-positive, false-positive, true-negative and false-negative results was constructed to determine the sensitivity, specificity, positive (PPV) and negative predictive values (NPV), where these were not directly presented.

Risk of bias and applicability were independently evaluated by two investigators (CB & KN) using the Quality Assessment of Diagnostic Accuracy Studies-2 (QUADAS-2) tool (14). It was anticipated that the heterogeneity of study designs, populations, assessment tools and reported outcomes was likely to preclude meta-analysis. Therefore, the authors made an ‘a priori’ decision to conduct a narrative synthesis.

## Results

### Study selection

**Figure 1.**
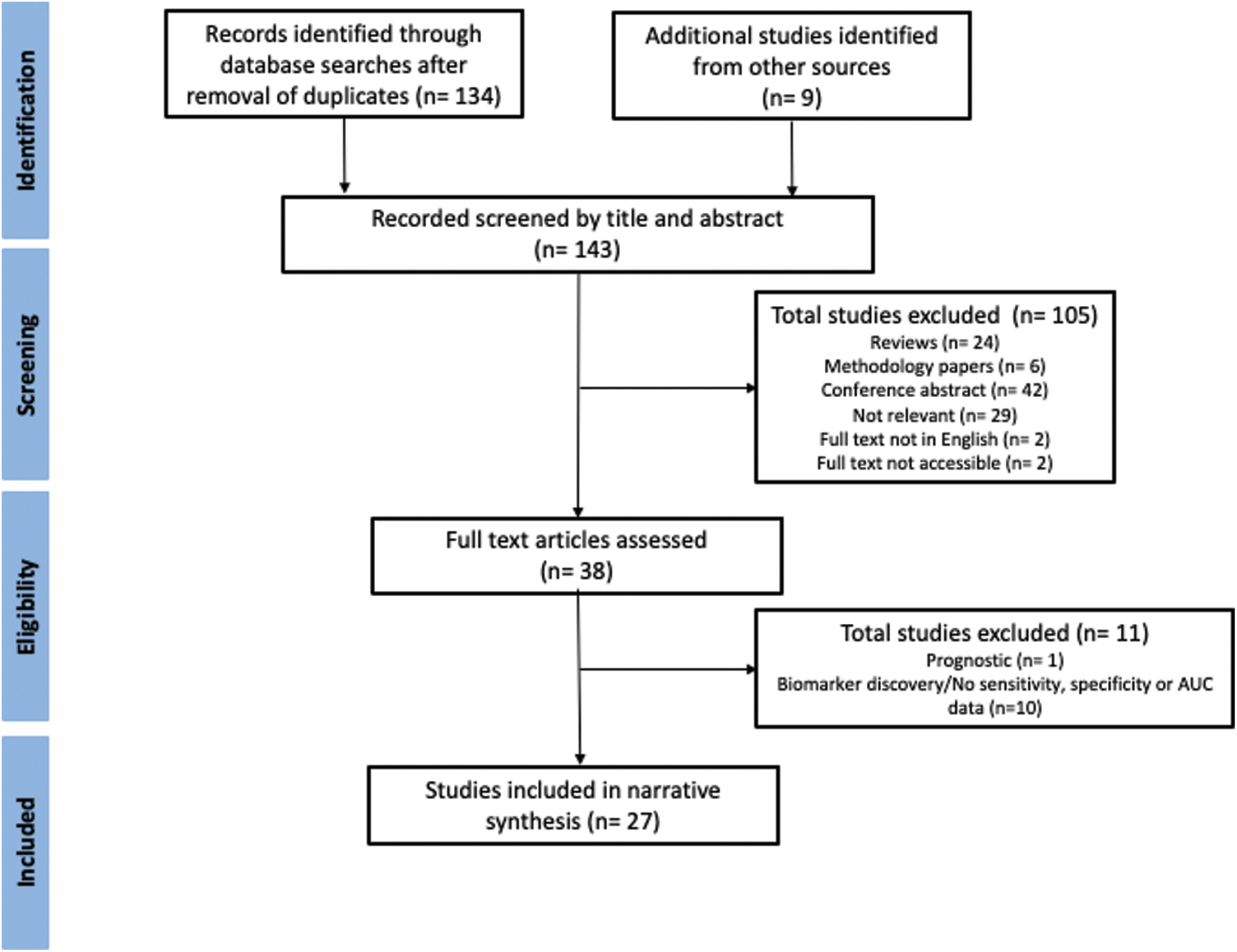
PRISMA flow diagram of study identification and selection.

The PRISMA flowchart summarizing the study selection process is shown in Figure 1. Database searches identified 134 unique articles. A further nine studies were identified through hand-searching the reference lists of relevant journal articles and conference abstracts. After initial abstract screening, 38 full-text manuscripts were assessed, of which 27 met the inclusion criteria and were included for narrative synthesis.

### Study characteristics

Characteristics of the included studies are summarized in Table 1. Most studies adopted a case-control design comparing urinary biomarkers in ovarian cancer patients and a control group. In most studies, the control group consisted of a combination of age-matched healthy volunteers and women with benign ovarian tumours or benign gynaecological disease. Most studies included mixed histopathological subtypes but four studies included serous ovarian cancer only (15–18). Nine studies originated from North America (15,19–26), ten from Europe (27–36), seven from Asia (16,17,37–41) and one from Australia (18).

**Table 1.** Study characteristic and diagnostic accuracy of urinary biomarkers for the diagnosis of ovarian cancer

Author, Year (Reference)	Country	Type of marker	Marker	Test platform	Study design	Urine collection	Histology	Stage I/II	Cases	Controls	Sens	Spec	PPV	NPV	AUC
Anderson, 2009 (19)	USA	Protein	Bcl-2	ELISA	Case control	NS	Mixed	13 (9%)	150	77 HD 161 Benign					0.90^^^
Ye, 2006 (20)	USA	Protein	EDN & osteopontin	SELDI-TOF MS	Case control	NS	EOC	55 (43%)	128	188	72	95*			
Hellstrom, 2010 (21)	USA	Protein	HE4	ELISA	Case control	Random	Mixed	15 (19%)	79	56	88.6	91.1	93.3	85.0	
Wang, 2011 (22)	USA	Protein	HE4	Microchip ELISA	Case control	NS	EOC	NS	19	20	89.5	90			0.94
Macuks, 2012 (27)	Latvia	Protein	HE4	ELISA	Case control	NS	NS	11 (48%)	23	55	78.3	75	56.3	89.1	0.86
Liao, 2015 (15)	USA	Protein	HE4	ELISA	Case control	NS	Serous	6 (7%)	92	187	51.1	94.7	82.5	79.7	
Fan, 2017 (35)	China	Protein	HE4	Electrochemi-luminescent immunoassay	Case control	NS	Mixed	11 (35%)	31	36	83.9	100	100	87.8	0.96
Zhou, 2015 (17)	China	Protein	HMGA1	ELISA	Case control	Morning whole-stream	Serous	NS	55	40					0.86 G1/2 0.88 G3
Tay, 1994 (36)	Singapore	Protein	CA125	ELISA	Prospective	Fasting morning	Mixed	NS	10	95	88.9	66.7	19.5	98.4	
Moore, 2009 (24)	USA	Protein	CA125	ELISA	Prospective	NS	Mixed	15 (22%)	67	166	33.2	90*			0.73
											17.4	95*			
											3.3	98*			
			Mesothelin								39.9	90*			0.71
											37.5	95*			
											24.6	98*			
Badgwell, 2007 (23)	USA	Protein	Mesothelin	ELISA	Case control	NS	Mixed	28 (20%)	139	127 HD	68	95			0.91
										155 Benign	49	95			0.81
Stockley, 2020 (28)	UK	Protein	MCM5	ELISA	Case control	Full void	Mixed	12 (46%)	26	58	61.5	75.9	53.3	81.5	0.68
Petri, 2009 (29)	Denmark	Protein	Fibrinogen beta fragment	SELDI-TOF MS	Case control	Non-fasting morning	EOC	10 (25%)	40	169					0.86
			Collagen alpha-1 fragment												0.76
			Fibrinogen alpha-1 fragment												0.74
			Three combined												0.88
Petri, 2010 (30)	Denmark	Protein	Four selected proteins inc. collagen alpha-1 fragment and trefoil factor 2	SELDI-TOF MS	Case control	Morning	EOC	5 (18%)	28	102					0.84
Sandow, 2018 (18)	Australia	Protein	Multiple proteins inc. four with AUC > 0.90; LYPD1	LFQ MS and PRM	Case control	Intra-operative from catheter	Serous	0 (0%)	20	20					0.92
			Mesothelin												0.91
			PTMA												0.92
			HE4 (WFDC2)												0.95
Lee, 2019 (39)	Korea	Protein	Panel of HE4, creatinine, CEA and transthyretin	Multiplexed immunoassay	Cohort	Fasting morning urine	Mixed	48 (30%)	158	125	93.7	70.6	78.7	90.6	0.94
Coticchia, 2011 (25)	USA	Protein	MMP-2, MMP-9	ELISA & substrate gel electrophoresis	Case control	NS	Mixed	0 (0%)	97	81	82^a^	75^a^	79.8	77.2	0.88^a^
Mu, 2016 (37)	Malaysia	Peptides	N-glycopeptides	SELDI-TOF MS	Case control	Morning mid-stream	NS	4 (100)	4	12	100	100	0	0	
Zhou, 2015 (16)	China	miRNA	miR-30a-5p	miRNA array and qPCR	Case control	Morning whole-stream	Serous	16 (41%)	34	25					0.86
			miR-6076												0.69
Slupsky, 2010 (26)	Canada	Metabolites		NMR Spectroscopy	Case control	NS	EOC	12 (24%)	50	62	98	99			
Zhang, 2013 (38)	China	Metabolites		UPLC-QTOF/MS	Case control	Fasting morning	Mixed	12 (30%)	40	116					0.73
Martinicky, 2015 (31)	Slovakia	Metabolites		Luminescence spectroscopy	Case control	Fasting morning	Mixed	13 (36%)	36	42 HD	91.7	100	100	99.3	
										35 Benign	86.1	77.1	79.5	84.4	
Niemi, 2018 (33)	Finland	VOCs		FAIMS	Case control	Fasting morning	Mixed	16 (48%)	33	18 HD	91.2	63.1			0.81
										18 Benign	91.5	51.4			0.77
Niemi, 2017 (32)	Finland	Polyamines	N,N-diacetylspermine	LC-MS/MS	Case control	Fasting morning	Mixed	18 (49%)	37	23	86.5	65.2	84.2	75	0.83
Paraskevaidi, 2018 (34)	UK	Chemical bonds		ATR-FTIR spectroscopy	Case control	Fasting morning	EOC	NS	10	10	100	96.3			
Giamougiannis2021 (35)	UK	Chemical bonds		Raman spectroscopy	Case control	Fasting	Mixed	33 (28%)	71 no NACT	307	45	85			
									45 NACT		100	87			
Giamougiannis2021 (36)	UK	Chemical bonds		ATR-FTIR spectroscopy	Case control	Fasting	Mixed	33 (28%)	71 no NACT	307	29	87			
									45 NACT		57	92			

Results of validation cohort reported.

*Pre-specified specificity.

Based on MMP-2 and MMP-9 in combination with age.

Abbreviations: ATR-FTIR, attenuated total reflection-Fourier transformation infrared; AUC, area under the curve; EOC, epithelial ovarian cancer; FAIMS, field asymmetric waveform ion mobility spectrometry; HD, healthy donor; LC-MS/MS, liquid chromatography-tandem mass spectrometry; LFQ MS, label-free quantitative mass spectrometry; NMR, nuclear magnetic resonance; NPV, negative predictive value; NS, not specified; PPV, positive predictive value; PRM, parallel reaction monitoring; qPCR, quantitative real-time PCR; SELDI-TOF MS, surface-enhanced laser desorption/ionization time-of-flight mass spectrometry; UPLC-QTOR/MS, ultra-high performance liquid chromatography-quadruple time-of-flight mass spectrometry.

Sample sizes of the studies range from 16 (four ovarian cancer patients, four healthy volunteers, four endometrial cancers and four cervical cancers) (39) to 423 (116 ovarian cancer patients and 307 benign gynaecological disease) (35,36). Most studies reported biomarker performance in the discovery cohort alone; only two validated their findings in an independent cohort (18,19).

### Normalization of data

Urinary protein biomarkers are frequently normalized to account for variations in urine flow rate across individuals. Four of the included studies were normalised to urinary creatinine levels (15,17,21,27), one normalised to total protein concentration (20) and one normalised using ratio of serum creatinine to urine creatinine (23). The remaining studies either did not normalise their data or did not specify whether normalization was performed, making it difficult to compare biomarkers across studies. Because diet, medication and alcohol can significantly affect the composition of metabolites within urine (42), seven studies evaluating urinary metabolites or organic compounds controlled for these potential confounders by collecting fasted urine samples (31–36,40).

### Risk of bias assessment

**Figure 2.**
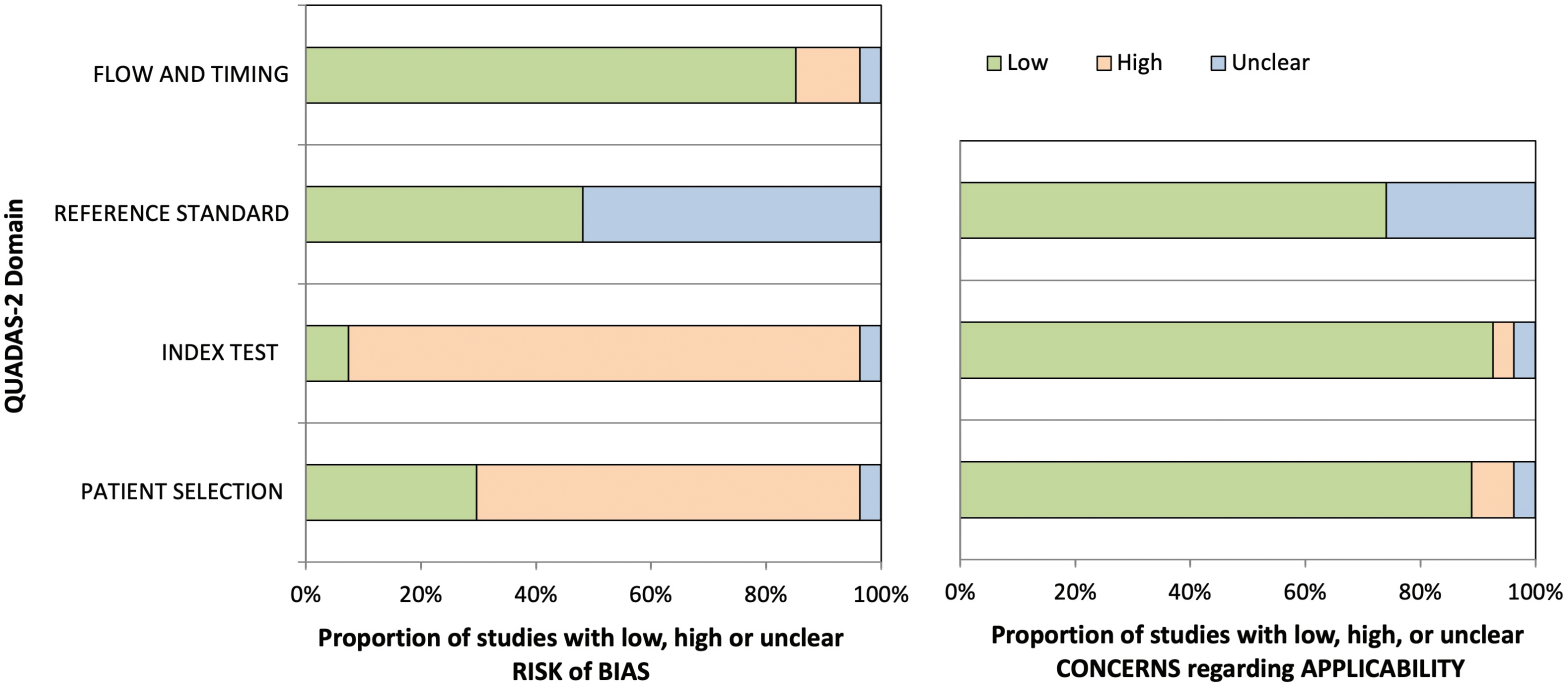
QUADAS-2 assessment of studies included in the systematic review.

A summary of the risk of bias assessment of the included studies is shown in Figure 2. Most studies incorporated a case-control design with groups consisting of urine from patients with ovarian cancer and control cases, indicating selection bias. Moore et al (24). was the only study to adopt a prospective design with blinding of the investigators to clinical and pathology results. None of the studies report a pre-planned statistical power calculation, owing to the exploratory nature of many of the studies. Similarly, 23 studies had a high risk of bias in determining the characteristics of the index test as they failed to pre-specify the threshold for a positive test.

### Diagnostic performance

Pragmatically, studies were included in this narrative synthesis if they reported either sensitivity/specificity or area under the ROC curve (AUC). Of the 27 studies included, seven reported AUC only (16–19,29,30,40), ten reported sensitivity and specificity only (15,20,21,26,31,34–36,38,39) and ten reported both measures of diagnostic performance (22–25,27,28,32,33,37,41). AUC is a measure of the overall performance of a diagnostic test, with an AUC of 1 denoting a perfect classifier. In this systematic review, six studies reported at least one biomarker with an AUC of >0.9 (18,19,22,23,37,41). To avoid overfitting, the biomarker should be validated in an independent patient cohort or through cross-validation models; however, only eleven studies appear to have done this (18–20,24,31,33–36,40,41).

### Protein and peptide biomarkers

Protein and peptide biomarkers were the most commonly tested urinary biomarker for the detection of ovarian cancer (*n* = 18). All included studies used either an immunoassay (*n* = 13) or mass spectrometry (*n* = 5) to quantify the protein/peptides of interest. Seven studies looked at multiple protein targets (18,20,24,25,29,30,41). Moore et al. (24). evaluated nine different biomarkers among women with benign gynaecological disease and ovarian cancer using individual immunoassays; however, only two biomarkers (CA125 and mesothelin) were quantified in urine samples, and these were not assessed in combination. Lee et al. (41). assessed 23 proteins using a multiplexed immunoassay, but found the combination of HE4, creatinine, carcinoembryonic antigen (CEA) and transthyretin (TTR) had the highest AUC of 0.94. Coticchia et al. (25). quantified levels of two metalloproteinases (MMP-2 and MMP-9) and neutrophil gelatinase-associated lipocalin (NGAL) in urine samples collected from women with ovarian cancer and healthy controls. They evaluated individual biomarkers and combinations of biomarkers for their diagnostic performance; but only reported the combination that most accurately classified ovarian cancer patients from controls. The best multivariate predictors of MMP-2, MMP-9 and age yielded an AUC of 0.881, with a sensitivity and specificity of 82% and 75%, respectively (25).

Three studies analysed the proteomic profile of urine collected from patients with benign and malignant ovarian masses using SELDI-TOF mass spectrometry and gel electrophoresis (20,29,30). Having identified higher levels of eosinophil-derived neurotoxin (EDN) and osteopontin in the urine of ovarian cancer patients, Ye et al. (20). developed ELISAs to test the diagnostic accuracy of these two biomarkers for ovarian cancer. Combining urinary EDN and osteopontin resulted in a sensitivity of 72% at 95% specificity to distinguish ovarian cancer from normal controls, compared with 47% and 63% for osteopontin and EDN alone (20). Despite having a cohort of patients with other cancers in this study, the sensitivity and specificity of these tests in differentiating ovarian cancer from other malignancies was not reported. In the first of two studies by Petri et al., 21 candidate urinary biomarkers were identified, of which fibrinogen alpha fragment, fibrinogen beta N-terminal fragment and collagen alpha-1 (III) fragment were the most discriminatory. Collectively, these biomarkers had a combined ROC AUC of 0.88 for differentiating ovarian cancer from benign ovarian masses. The ROC AUC increased to 0.96 when all three biomarkers were combined with serum CA125 (29). In the latter study, designed to validate the candidate biomarkers in an independent patient cohort, only four of 21 biomarkers were discriminatory. The authors suggested that differences in diagnostic accuracy of the biomarker panel between initial and validation cohorts may be explained by selection of surrogate biomarkers from exoprotease activities, over-fitting of the data or a statistical type two error in the original study. The ROC AUC for the urinary biomarker panel in latter study was 0.84 (30).

Of the nine studies looking at a single protein biomarker, five reported the diagnostic accuracy of urinary HE4 (15,21,22,27,37). Three of these studies (15,21,27) have previously been pooled with four other studies (not included in this systematic review as full-text not available in English) in a meta-analysis of 413 cases and 573 controls, to give a combined sensitivity of 76%, specificity of 92% and AUC of 0.93 (43). Across the individual studies included here, three studies had a sensitivity and specificity of greater than 80% (21,22,37). However, there is significant heterogeneity between these studies, particularly with regards to sample size, included histological subtypes and the percentage of patients with early-stage disease. Wang et al (22). reported the highest sensitivity and specificity (89.5% and 90%, respectively) using a novel microchip ELISA coupled with a cell phone to quantify HE4. However, this is the smallest of the studies evaluating urinary HE4 with only 19 cancer cases included.

Two studies explored the diagnostic accuracy of urinary CA125 (24,38) and mesothelin (23,24). Moore et al. cross-validated logistic regression models using a leave-one-out approach to obtain average sensitivities at set specificities of 90%, 95% and 98% for each biomarker. At a specificity of 90%, sensitivity was less than 40% for both markers (24). In an earlier study, Tay et al. reported a sensitivity of 88.9% and specificity of 66.7% for detecting ovarian cancer using urinary CA125 (38). However, this study only included 10 cases and did not specify FIGO stage.

A further three studies have assessed Bcl-2, HMGA1 and MCM5 as individual protein biomarkers (17,19,28). All three studies utilized ELISAs to quantify the protein of interest. Anderson et al. reported an AUC of 0.93 using urinary Bcl-2 to differentiate between ovarian cancer cases and healthy donors; however, this test was less accurate at discriminating between benign and malignant disease. Evaluation of urinary HMGA1 in a study limited to serous ovarian cancer yielded AUCs of 0.86 and 0.88 for grade 1/2 and grade 3 cancers, respectively (17). The performance of this biomarker has yet to be validated in an independent cohort. The sensitivity and specificity of urinary MCM5 is not currently high enough to warrant further testing of this biomarker for ovarian cancer detection (28).

Using SELDI-TOF mass spectrometry, Mu et al. (39). investigated glycosylated peptides in endometrial, cervical and ovarian cancers. In this small study of 16 samples, which included four-stage I/II ovarian cancers, the urinary glycopeptide peak *m*/*z* 1201 was able to differentiate ovarian cancer from non-ovarian cancer (mixed cohort of healthy donors, cervical and endometrial cancers) with a sensitivity and specificity of 100%. Although these results suggest that the urinary glycopeptide *m*/*z* 1201 could serve as a potential biomarker for early detection of ovarian cancer, this requires extensive validation in an independent and clinically representative population.

### Epigenetic biomarkers

Only one study investigated microRNAs (miRNAs) in urine (16). MiRNAs are involved in post-transcriptional regulation of gene expression, making them attractive biomarkers in cancer. Zhou et al (16). first employed miRNA arrays to identify differentially expressed targets, and then quantified by real-time qPCR. Thirty-seven miRNAs were downregulated but only miR-30a-5p was upregulated in the urine samples of ovarian serous adenocarcinoma patients compared to healthy controls. A strength of this study is that miR-30a-5p levels were also determined in tissue samples from patients and controls to ensure urinary miR-30-5p was derived from ovarian tumour tissue. The ability of urinary miR-30a-5p and miR-6076 levels to distinguish ovarian serous adenocarcinoma patients from healthy volunteers was determined, with miR-30a-5p showing very good discrimination with an AUC of 0.862 (16). As with many of the studies discussed in this narrative review, these results are yet to be externally validated.

### Metabolites and organic compounds

Four studies describe the diagnostic accuracy of urinary metabolites (26,31,32,40). We identified further studies that reported alterations in the metabolomic profile of patients with ovarian cancer compared to controls, but did not evaluate diagnostic accuracy (44,45). The analytic platforms used for metabolite detection differed across each study and included nuclear magnetic resonance (NMR) spectrometry, ultra-high performance liquid chromatography-quadruple time-of-flight mass spectrometry (UPL-QTOF/MS), liquid chromatography-tandem mass spectrometry (LC-MS/MS) and luminescence spectroscopy. None of the included studies validated the results of urine metabolite profiling using tissue. Profiling of urinary metabolites identified discriminatory metabolites that are able to distinguish patients with ovarian cancer from healthy donors and benign ovarian tumours. Up- or down-regulated urinary metabolites in ovarian cancer relate to perturbed glycolysis, the tricarboxylic acid (TCA) cycle, amino acid and nucleotide metabolism. Of note, succinate was the only metabolite to be reported in two studies, and showed inconsistent results with up-regulation in one study and down-regulation in the other (26,40). Differences in analytic platforms, specimen collection and preparation may account for this. Both Slupsky et al. (26). and Martinicky et al. (31). reported a sensitivity and specificity of >90% for discriminating ovarian cancer from healthy volunteers, based on metabolomic profile. However, sensitivity and specificity decreased to 86.1% and 77.4% respectively, when discriminating benign and malignant ovarian tumours (31). One study specifically evaluated urinary polyamines in women with adnexal masses (32). In this study, only urinary *N*^1^,*N*^12^-diacetylspermine showed potential as a biomarker with elevated levels in malignant vs. benign tumours. With regards to diagnostic accuracy, DiAcSpm had a higher sensitivity (86.5%) but lower specificity (65.2%) for distinguishing benign and malignant ovarian tumours when compared to CA125 using the standard cut-off value of 35 kU/l (32).

Volatile organic compounds (VOCs), generated through metabolism of cells and excreted through exhaled breath or body fluids, are currently attaining traction as cancer biomarkers. VOCs are thought to reflect biochemical changes within the body as a result of biological activities such as oxidative stress, inflammation and apoptosis. Initial interest in the potential role of VOCs in cancer detection evolved from early reports of the ability of trained sniffer dogs to identify cancer. Artificial olfaction technologies have now been developed to qualitatively analyse VOCs. We identified one study in the literature that utilized field asymmetric waveform ion mobility spectrometry (FAIMS) to distinguish urine of women with ovarian cancer from benign ovarian tumours and controls (33). FAIMS had a 91.2% sensitivity and 63.1% specificity for differentiating controls from ovarian cancer.

Vibrational spectroscopy is a novel technique that provides a direct measurement of chemical bonds within a biological sample. Infrared (IR) and Raman spectroscopy have been used extensively in cancer diagnostics across multiple tissue types and biofluids, including plasma/serum, urine and ascitic fluid. To date, two studies have utilized attenuated total reflection-Fourier transform infrared (ATR-FTIR) spectroscopy and one study has utilized Raman spectroscopy to analyse urine samples from women with ovarian cancer and controls (34–36). Paraskevaidi et al. assessed the performance of urine in a cohort comprising of 10 ovarian cancer patients and 10 healthy controls and found sensitivity, specificity and accuracy of 100%, 97.5% and 98.3%, respectively, employing the PCA-SVM classification algorithm. The top six discriminatory peaks were predominantly attributed to proteins and nucleic acids (34). In a prospective study of 307 patients with benign gynaecological conditions and 116 with ovarian cancer, including women who had received neoadjuvant chemotherapy, urine demonstrated poor sensitivity for diagnosis of ovarian cancer in chemo-naïve patients using both ATR-FTIR spectroscopy (29%) and Raman spectroscopy (45%) (35,36). This study, however, failed to account for the impact of potential confounders.

## Discussion

Studies on urinary biomarkers for ovarian cancer are relatively sparse. The majority of studies have evaluated single protein assays such as HE4 or mesothelin; however, more recently, metabolic changes and circulating microRNAs have been assessed as potential urinary biomarkers. Only six studies achieved a sensitivity and specificity of >90% and/or an AUC > 0.9 (Table 1) (18,22,26,34,39,41). However, three of these studies sampled apparently healthy women as the control group rather than women with benign ovarian masses (22,26,34). Although urinary Bcl-2 looks promising with an AUC of 0.90 reported in the validation cohort, this biomarker is not specific to ovarian cancer as it is also over-expressed in lymphoma, colorectal and lung cancer, and is therefore unlikely to be useful in clinical practice (19). The ability of N-glycopeptides to correctly identify all four cases of early ovarian cancer in the pilot study by Mu et al. (39). is intriguing but requires validation in a much larger cohort. ‘Omics’ approaches such as metabolomics also look promising, with urinary metabolomic profiling achieving good separation between breast and ovarian cancer (26,44). However, Woo et al (44). were unable to discriminate between ovarian and cervical cancer specimens, indicating that further studies are required to determine the specificity of the metabolites and their ability to discriminate between different types of gynaecological cancers (46).

In this systematic review, the diagnostic accuracy of urinary biomarkers varied significantly. The sensitivity and specificity of single protein/peptide assays ranged from 3.3 to 100% and 66.7 to 100%, respectively. Most protein/peptide biomarkers were only assessed in one or two studies, with the exception of HE4. HE4 had a sensitivity of 51.1–89.5% and specificity of 75–100% across five independent studies. Such variation in diagnostic accuracy may be explained by differences in patient selection, urine collection and storage, histological subtypes and assay factors. Currently, serum CA125 is utilized to predict the presence of ovarian malignancy in women with a pelvic mass. A 2013 meta-analysis reported that serum CA125 had an overall sensitivity of 79% (95% CI, 77–82%) and a specificity of 78% (95% CI, 76–80%) in detecting ovarian cancer (47). The sensitivity and specificity of any urinary biomarker would therefore need to be higher than that achieved by serum CA125 alone to warrant implementation as a triage test prior to surgery (48).

There is a growing interest in urinary biomarkers for cancer early detection reflecting advances in technology that enable their detection at ever-lower concentrations (49–54). Urinary biomarkers may derive from the renal excretion of systemic biomarkers or the passive contamination of urinary flow with tumour debris shed from the lower genital tract (42). Simple, non-invasive, painless, cost-effective and convenient were the most important attributes of a new cancer test for patients, clinicians and the general public in the recently completed James Lind Alliance Priority Setting Partnership for Detecting Cancer Early (48). Urinary protein biomarkers detected, for example, by lateral flow technology could offer a point of care test for symptomatic women presenting to their GP with suspected ovarian cancer. A rapid result could provide quick reassurance for test negative women whilst expediting specialist referral for those who test positive. A urine test also lends itself to home-based self-sampling with postal return to the laboratory for asymptomatic women at increased risk of ovarian cancer (eg BRCA1/2 pathogenic variant carriers), for whom repeat sampling at regular intervals could be important for early detection. Urine may be an invaluable source of early-stage disease biomarkers which may only be detectable in blood for a limited period of time and even then only present at very low concentrations (12). A number of the studies in this review compared biomarker levels in matched serum and urine from the same donors. Of note, Badgwell et al. (23). found a higher proportion of patients with early-stage disease were detected with urine (42%) than serum assays (12%). Despite clear potential, there are also several drawbacks to using urine as a source of biomarkers. First, systemic biomarkers may not be excreted in urine, especially in early-stage tumours. Second, biomarkers originating from natural tumour shed may be unreliable in their detection. Third, urine protein and metabolite concentrations may vary with exogenous factors that need to be controlled for, including demographic variables, diet, medications and fluid intake.

There are a number of limitations to our systematic review. First, many of the studies exploring urinary biomarkers for ovarian cancer detection have been small pilot studies with fewer than 50 cases. Most of the studies recruited patients from a single cancer centre limiting the applicability to the general population. Furthermore, the lack of validation cohorts and cross-validation modelling in many of the studies may have led to potential over-fitting in the interpretation of results. Second, there is significant heterogeneity between studies regarding study population, methodology and analytic platforms. Pre-analytical variables such as timing of urine collection, volume collected, sample preparation and freeze-thawing protocols were not clearly reported in many of the studies. Some studies did not specify how they normalised urine concentration. There were also notable variations in the definition of the control groups between studies, which greatly influences the estimated specificity of a particular biomarker. This was evident in three studies that reported the sensitivities and specificities for differentiating cancer cases from healthy donors and benign disease (23,31,33). Asymptomatic screening of the general population for early detection of cancer should be clearly distinguished from diagnostic work-up of patients with a known pelvic mass. Third, there was inadequate reporting of clinical parameters including cancer stage in several studies, making it difficult to evaluate the performance of these urinary biomarkers in the diagnosis of early-stage cancer. For studies that did report cancer stage, the majority of patients had advanced disease. Finally, only articles published in English were included in this review, and therefore we may have overlooked some novel biomarkers.

In conclusion, there is a dynamic and growing field of urinary biomarker research for the detection of ovarian cancer. Urinary proteins, metabolites and microRNA have all been evaluated as potential ovarian cancer biomarkers. Currently, the most promising biomarkers appear to be those that interrogate metabolomic pathways and organic compounds, or quantify multiple proteins. Unfortunately, many of the studies presented in this review are only at the biomarker discovery phase, and there is insufficient evidence to support their use in routine clinical practice. Future efforts should focus on conducting large, prospective, multi-centre studies to assess the true potential of these biomarkers.

## Data Availability

The authors confirm that the data supporting the findings of this study are available within the article.

## Abbreviations

CA125: Cancer antigen 125
HE4: Human epididymis 4
ROC: receiver operating characteristics
TVS: Transvaginal ultrasonography
